# Effect of electroacupuncture versus pelvic floor muscle training plus solifenacin for moderate and severe mixed urinary incontinence in women: a study protocol

**DOI:** 10.1186/1472-6882-14-301

**Published:** 2014-08-15

**Authors:** Baoyan Liu, Yang Wang, Huanfang Xu, Yuelai Chen, Jiani Wu, Qian Mo, Zhishun Liu

**Affiliations:** Guang’anmen Hospital, China Academy of Chinese Medical Sciences, No.5 Beixiange Street, Beijing, Xicheng District China; Shanghai University of Traditional Chinese Medicine, 1200 Cailun Road, Shanghai, Pudong China

**Keywords:** Electroacupuncture, Pelvic floor muscle training, Solifenacin, Mixed urinary incontinence

## Abstract

**Background:**

In women with mixed urinary incontinence, pelvic floor muscle training and solifenacin is the recommended conservative treatment, while electroacupuncture is a safe, economical and effective option.

**Methods/Design:**

In this prospective, multi-center, randomized controlled trial, five hundred women with mixed urinary incontinence, from 10 centers will be randomized to receive either electroacupuncture or pelvic floor muscle training plus solifenacin. Women in the acupuncture group will receive electroacupuncture for 3 sessions per week, over 12 weeks, while women in the control group will receive pelvic floor muscle training plus solifenacin (5 mg once daily) for 36 weeks. The primary outcome measure is the proportion of change in 72-hour incontinence episode frequency from baseline to week 12. The secondary outcome measures include eleven items, including proportion of participants with ≥50% decrease in average 72-h incontinence episode frequency, change from baseline in the amount of urine leakage and proportion of change from baseline in 72-h incontinence episode frequency in week 25–36, and so forth. Statistical analysis will include covariance analysis, nonparametric tests and t tests.

**Discussion:**

The objective of this trial is to compare the efficacy and safety of electroacupuncture versus pelvic floor muscle training plus solifenacin in women with moderate and severe mixed urinary incontinence.

**Trial registration:**

ClinicalTrials.gov Identifier: NCT02047032

## Background

The International Urogynecological Association/International Continence Society defines mixed urinary incontinence (MUI) as “the complaint of involuntary loss of urine associated with urgency and also with effort or physical exertion or on sneezing or coughing” [1]. MUI occurs when symptoms of both stress urinary incontinence (SUI) and urgency urinary incontinence (UUI) are present. According to the literature, the prevalence of MUI in women range from 16 to 61% [1–3]. In China, the prevalence is 9.4% [4].

The treatment of MUI is challenging because the symptoms of SUI and UUI occur concurrently. Management options include absorbent products, behavioral modification, medical management, and surgery. Behavioral therapy (such as lifestyle modification, bladder training and pelvic floor muscle training (PFMT)) should be considered as a first-line option for all women with MUI [5, 6]. Although recommended for MUI, PFMT is the most commonly recommended physical therapy for women with SUI [7]. Its benefit seems to be greater in women with SUI alone as compared with women with mixed SUI and UUI [8]. Thus, for the treatment of MUI, drug therapy is usually used in combination with PFMT [9].

Solifenacin is a M3 receptor selective antimuscarinic agent, launched in 2005 for the treatment of overactive bladder. It relieves the symptoms of frequency, urgency, nocturia and urgency incontinence [10]. In a meta-analysis of seven studies, the mean and median reduction in the number of urgency episodes with solifenacin was greater than with several other agents such as oxybutynin, tolterodine, trospium, and darifenacin [11]. For this study, the combination of PFMT and solifenacin will serve as a control against electroacupuncture.

Acupuncture has a long history and is a widely used conservative treatment option for many diseases, including urinary incontinence. A literature review showed that acupuncture improves symptoms of both SUI and UUI, and it seems to have long-term benefits [12–14]. Based on this, acupuncture is a reasonable treatment option for MUI.

This is a protocol for a prospective, multi-center, randomized controlled trial. The primary objective of the study is to compare the effect of electroacupuncture (EA) versus PFMT plus solifenacin in patients with MUI. The secondary objective is to ascertain whether EA is effective for another 12 to 24 weeks after the therapy.

## Methods and design

### Study design

This study is a prospective, randomized controlled trial and will be conducted from March 2014 to August 2015 across 10 hospitals in China^a^.

**Figure 1:**
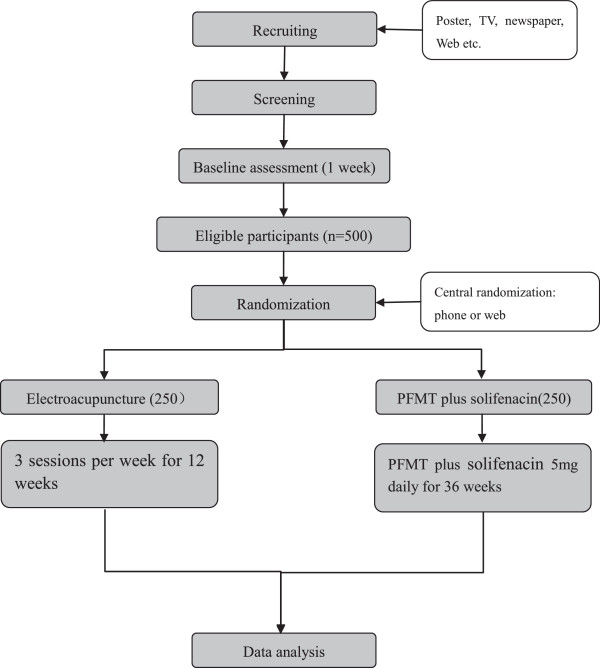
**The flowchart.**

Participants with urinary incontinence will be recruited through advertisements in newspapers, on television, on websites and posters. The diagnosis of incontinence type and severity will be made by a gynecologist or a urologist [15]. After obtaining informed consent, patients with moderate MUI will receive a 1-week baseline assessment, including a 72-hour voiding diary, a 1-hour pad test, routine urinalysis, a urine flow rate assessment and a bladder ultrasound. Randomization will be performed centrally by the Clinical Evaluation Center of the China Academy of Chinese Medical Sciences in Beijing, and eligible participants will be randomized to the EA group or the control group. A randomization number will be sent immediately to the acupuncturist by phone or online. The statisticians, outcome assessors, the gynecologists and urologists will be blinded to the allocation. The flowchart of the trial is presented in Figure 1. The trial protocol is in accordance with the principles of the Declaration of Helsinki and has been approved by the review board and ethics committee of the participating hospitals (Ethics approval number: 2013EC125-01).

### Participants

Five hundred participants will be needed in this trial. Patients meeting the diagnostic criteria of concomitant SUI and UUI will be diagnosed as mixed urinary incontinence.

Diagnostic criteria of SUI: 1) involuntary loss of urine on effort or physical exertion, or on sneezing or coughing; 2) positive in urinary stress test.

Diagnostic criteria of UUI: unwanted urine loss that happens shortly after the sudden, intense desire to urinate.

### Inclusion criteria

Participants who meet the following criteria will be included: 1) confirmation of mixed urinary incontinence; 2) aged 35–75 years; 3) moderate and severe urinary incontinence with a urinary incontinence severity index between 3 and 9 [16] 4) a history of urinary incontinence for at least 3 months and a 72-hour incontinence episode frequency (IEF) ≥2 at baseline; 5) volunteer to join this research and give informed consent prior to receiving treatment.

### Exclusion criteria

Participants with any of the following conditions will be excluded: 1) stress urinary incontinence, urgency urinary incontinence, overflow incontinence or neurogenic bladder; 2) the use of medication for urinary incontinence, medication that may affect bladder function, or the use of any non-drug therapy (such as electric stimulation, bladder training and pelvic floor muscle training) in the prior month; 3) symptomatic urinary tract infection and non-functional urologic disease; 4) history of surgery for urinary incontinence or to the pelvic floor (including hysterectomy); 5) second-degree or greater pelvic organ prolapse; 6) residual urinary volume (RUV) >30 mL; 7) maximum flow rate (Qmax) <20 mL/s; 8) allergy to solifenacin or contraindications to muscarinic antagonists (such as urinary retention, gastric retention, myasthenia gravis, ulcerative colitis and angle closure glaucoma); 9) diseases affecting the functioning of the lower urinary tract (such as uncontrolled diabetes, multiple sclerosis, Alzheimer’s disease, Parkinson’s disease, spinal injury, cauda equina injury and multiple system atrophy); 10) serious cardiovascular, pulmonary, cerebral, liver, kidney, blood or psychiatric disease and cognitive impairment; 11) severe renal dysfunction or moderate hepatic dysfunction with concomitant strong CYP3A4 inhibitor use (such as ketoconazole); 12) limited or no ability to walk up and down stairs and running; 13) poor compliance with EA, pelvic floor muscle training or medication; 14) pregnancy, lactation or within 12 months postpartum; 15) a cardiac pacemaker, a metal allergy, or a severe needle phobia; 16) volunteer in other trials.

### Intervention

All participants will be advised of lifestyle modification, including: 1) weight management (participants with a BMI ≥30 would be advised to lose weight); 2) fluid intake (1.5 to 2 L in 24 hours) 3); caffeine reduction. Therapies other than the treatment regimen are prohibited during the trial.

#### Treatment group

BL33 point and BL35 point of both sides are used. For BL33, the needle will be inserted at the third sacral foramen at an angle of 30–45° and to a depth of 50–60 mm. The needle will be manipulated with an even lifting, thrusting and twisting method and a sense of soreness and distention will radiate to the perineum or the anus. For BL35, the needle will be inserted upward and outward and then manipulated with an even lifting, thrusting and twisting method and a sense of soreness and distention will radiate to the perineum or the anus. An electric stimulator will be placed on the pair of points with a spare-dense wave, 10/50 Hz, 0.1–5.0 mA. The current intensity will be increased to maximum tolerance and then reduced to a bearable limit. Following a review of the Chinese literature published in the last 10 years, together with the results of a phase I trial and expert consensus, all patients will receive 36 acupuncture sessions (3 sessions per week for 12 weeks), with each session lasting 30 minutes and administered every other day.

#### Control group

The control group will receive PFMT plus solifenacin for a period of 36 weeks. Solifenacin (Astellas Pharma Europe B.V.) will be taken at a dose of 5 mg once daily, before or after a meal. PFMT will include intensive exercises conducted in hospital and home exercises. Intensive exercises will be done once a week for the first 12 weeks and once every four weeks for weeks 13–36. Home exercises will be done three times daily for 36 weeks. The intensity of the exercises will conform to the National Institute for Health and Clinical Excellence (NICE) [17] guidelines.

### Outcome measures

In this study, there is one primary outcome and eleven secondary outcomes. These are presented in Table 1. Safety evaluation for EA will be based on events which include fainting, severe pain, hematoma, local infection, and any feelings of discomfort. Any adverse event resulting from EA or adverse drug reaction to solifenacin will be recorded.

**Table 1:** **Outcome measures**

	Outcome measure	Time frame	Description	Statistics
Primary outcome	Proportion of change from baseline in 72-h IEF over week1-12	Week 1-12	The average 72-h IEF is calculated based on a 72-h bladder diary. The average 72-h IEF of week 1-12 is calculated by averaging the IEF of week 2, 4, 6, 8, 10, 12.	Covariance analysis or nonparametric test
Secondary outcome	Proportion of change from baseline in 72-h IEF	Week 13–36, week 1–4, week 5–8, week 9–12, week 13–24, week 25–36	Calculated as the same way as the primary outcome. But this outcome will be assessed at different time point.	A t test or nonparametric test
	Proportion of participants with ≥50% decrease in average 72-h IEF	Week 1–12, 13–24, 13–36	Count the number of cases with the reduction of average 72-h incontinence episode frequency ≥50% and divide t by the number of participants at baseline.	A chi-square test or nonparametric test
	Change from baseline in 72-h IEF	Week 1–12, 13–24, 13–36	The average 72-h IEF (urinary incontinence, stress urinary incontinence and urgency urinary incontinence respectively) is calculated based on a 72-h bladder diary. For example, the average 72-h incontinence episodes from the 13th to 36th week equal to the sum of 72-h incontinence episodes of the 16th, 20th, 24th, 28th, 32nd, 36th weeks divided by 6.	A t test or nonparametric test
	Change from baseline in the International Consultation on Incontinence Questionnaire-Short Form score	Baseline, week 1–12, 13–36	The score of week 1–12 equals to the sum score of week 4, 8, 12 divided by 3.	A t test or nonparametric test
	Change from baseline in the amount of urine leakage	Baseline, week 4, week 12	The amount of urine leakage was tested by 1-h pad test.	A t test or nonparametric test
	Change from baseline in average 72-h urgencies/urination/nocturia episodes	Baseline, week 1–12, 13-36	The average 72-h urgencies/urination/nocturia episodes within a period equal to the sum of urgencies/urination/nocturia episodes (counted according to the urinary diary) divided by the number of weeks recorded.	A t test or nonparametric test
	Change from the baseline in the number of pads used in one week	Baseline, week 1–12, 13–36	The mean of pads used in one week equals to the sum of pad used in a period divided by the number of weeks within this period.	A t test or nonparametric test
	Proportion of change from the baseline in 72-h IEF of subgroups	Week 1–12, 13–36	Stratified by incontinence severity/types of mixed urinary incontinence/age at the baseline, the percent of change in 72-h incontinence episode frequency will be analyzed.	A covariance analysis or nonparametric test
	Satisfaction degree	Week 12, 36	A questionnaire will be done by participants to evaluate whether they are satisfied with the treatment	A chi-square test or nonparametric test
	Patient global impression improvement	Week 12, 36	Participants will be asked to finish one item evaluating their present condition.	A chi-square test or nonparametric test
	Electroacupuncture acceptance assessment	Treatment session 1, 18, 36	After the 1st, 18th and 36th session, the acceptance of electroacupuncture will be tested within 5 minute with a 5-point scale (‘0’ means very difficult to accept and ‘4’ means accept easily). Mean of scores of the three times will be calculated.	A chi-square test or nonparametric test

### Data collection and quality control

Two researchers will input data independently using the Remote Data Capture (RDC) software. A Data Verification Plan (DVP) will be established to review the data after input. Two data managers with a medical background will perform coding for the medical history, adverse events and drug combinations. After a data review, the data will be submitted to the statistician for final analysis.

To guarantee the quality of the trial, a rigorous methodology will be followed. Before commencing the trial, experts in different fields will be invited to review and revise the protocol, and staff from the 10 trial centers will undergo training. A 3-level monitoring system will be established to check the performance of the trial periodically. Outcome assessments, completion of case report forms and data management will be closely supervised.

### Sample size and statistical analysis

Sample size will be based on the primary outcome. According to our pilot trial and review of the literature [15, 18–20], the proportion of change in 72-hour IEF from baseline for EA and PFMT plus solifenacin, is 57 and 60% respectively. To assess non-inferiority between the treatment and control groups, a sample size of 242 for each group will be sufficient, with a one-sided 5% level of significance, a power of 80% and allowing for a 15% dropout. This will exceed the non-inferiority margin of 15% [21]. Thus, in this trial, we will aim to recruit 250 participants in each group.

Data from the 10 centers will be pooled, and the Clinical Evaluation Center of China Academy of Chinese Medical Sciences in Beijing will conduct the statistical analysis, using the SAS 9.1.3 (SAS Institute, Cary, NC, US) and SPSS Ver.13.0 (SPSS Inc., Chicago, IL, USA) software. All statistical analyses will be two-sided tests except for the primary outcome. The level of significance will be established at 0.05. Continuous data will be represented by the mean, standard deviation, median, minimum value, and maximum value; categorical data will be represented by percentages. For comparison with the baseline, a t-test or nonparametric test will be used for continuous data, and nonparametric tests for categorical data. For comparison of two independent samples a t-test or nonparametric test will be used for continuous data, and a chi-square test or the Fisher exact test will be used for categorical data. The primary outcome is the proportion of change from baseline in 72-h IEF. Analysis of covariance (for normal distributed data) or nonparametric tests (for abnormal distributed data) will be used. To detect the center effect, a covariance model will be used. Further analysis will be done if there is a center effect. Analysis methods for secondary outcomes are presented in Table 1. For safety analysis, incidence of adverse events will be compared between the two groups using the chi-square test or the Fisher exact test.

## Discussion

In this non-inferiority, randomized controlled trial, we intend to compare the efficacy of EA versus PFMT plus solifenacin. Although PFMT is effective for the treatment of MUI, long-term adherence is necessary [6, 22]. Additionally, compliance with muscarinic antagonists is poor; owing to its side effects (such as dry mouth, constipation and blurred vision) [23]. Acupuncture is a non-toxic, economical intervention with minimal adverse effects [24], which has been shown to remain effective even for a few months after the therapy [25]. If the efficacy of EA is non-inferior to PFMT plus solifenacin, it may be a reasonable option in patients with MUI, requiring a conservative approach. One of the limitations in this trial design, is the non-blinding of the participants and acupuncturists. Due to the characteristics of acupuncture and the control (drug and PFMT), it is impossible to blind the participants and acupuncturists. However, we will rigorous methodology in other part of the study.

### Trial status

Twelve participants have been recruited.

### Endnote

^a^Guang’anmen Hospital; Dongzhimen Hospital (Beijing); West China Hospital (Chengdu); Yantai TCM Hospital (Yantai); Hengyang Hospital of Hunan University of Chinese Medicine (Hengyang); The First Hospital of Hunan University of Chinese Medicine (Changsha); Hubei Provincial Hospital of TCM (Wuhan); Jiangsu Province Hospital of TCM (Nanjing); Shanxi Province Hospital of TCM (Xi’an) and The Hiser Healthcare (Qingdao).
